# Highlight: Exceptions Are the Rule in Sex Determination

**DOI:** 10.1093/gbe/evaa092

**Published:** 2020-06-03

**Authors:** Casey McGrath

For nearly a century, biologists have modeled the evolution of sex chromosomes—the genetic instructions that primarily determine whether an individual will develop into a male or female (or a certain mating type)—resulting in an impressive theoretical framework. Now, thanks to the publication of genomic data from a wide variety of nonmodel organisms, these theories are being tested against empirical evidence from nature—often with surprising results. In a new review in *Genome Biology and Evolution*, Benjamin Furman, Judith Mank, and colleagues detail the surprising number of exceptions to the purported rules of sex chromosome evolution theory, revealing the potential limitations to our understanding of chromosome-based sex-determination systems (Furman et al. 2020).

It is often taken for granted that sexual reproduction requires the participation of two individuals of different sexes—generally males and females in the case of plants and animals. Although there are numerous exceptions, such as hermaphroditic species of plants, worms, and fish, separate sexes are indeed seen frequently across eukaryotes, especially among animals. An individual’s sex may be determined in various ways in different species; although some systems are driven by environmental or social cues, sex is often determined by the presence or absence of a specific sex chromosome—a discrete unit of DNA carrying one or more genes that initiate the development of male- or female-specific characteristics.

Since the first discovery of sex chromosomes by Nettie Stevens in 1905, who noted that male but not female mealworms carried one chromosome smaller than the rest, a considerable body of theory predicting the forces that govern sex chromosome evolution has arisen. The generally accepted model for sex chromosome evolution goes like this: 1) A genetic variant arises on an autosome (a nonsex chromosome) and specifies either the male or the female sex; 2) the acquisition of sexually antagonistic alleles, which are advantageous in one sex but disadvantageous in the other, near the new sex-determining gene favors the suppression of recombination, allowing, for example, male-beneficial traits to be inherited in males only; 3) genes are eventually lost from the new sex chromosome, leading to imbalances in protein expression in one sex; and 4) mechanisms evolve to correct the imbalances in gene dosage. Notably, this model assumes a successive and irreversible progression from autosome to fully differentiated, dosage-compensated sex chromosome.

In reviewing the newly emerging literature from a broad range of organisms, Furman, Mank, and coauthors were stunned at the number of findings that deviate from the above rules. According to Mank, “All of us in the field know that although much of this theory has been upheld in many organisms, there are notable exceptions. Until we compiled this review, we did not really have a sense of how common the exceptions are, or how much diversity there is in sex chromosome evolution.”

These exceptions call into question how broadly applicable each of the canonical steps in sex chromosome evolution may be. For example, sex chromosomes in some insects are derived not from autosomes but from nonessential selfish chromosomes called B chromosomes or from bacteria-derived DNA that has made its way into the nuclear genome. Moreover, rather than sexual antagonism driving recombination suppression, recombination may instead be initially inhibited due to the movement of transposable elements or other epigenetic changes to young sex chromosomes. In addition, global dosage compensation of sex chromosomes (as found in humans) appears to be the exception rather than the rule, with most species showing incomplete, gene-by-gene compensation mechanisms.

**Fig. 1.**
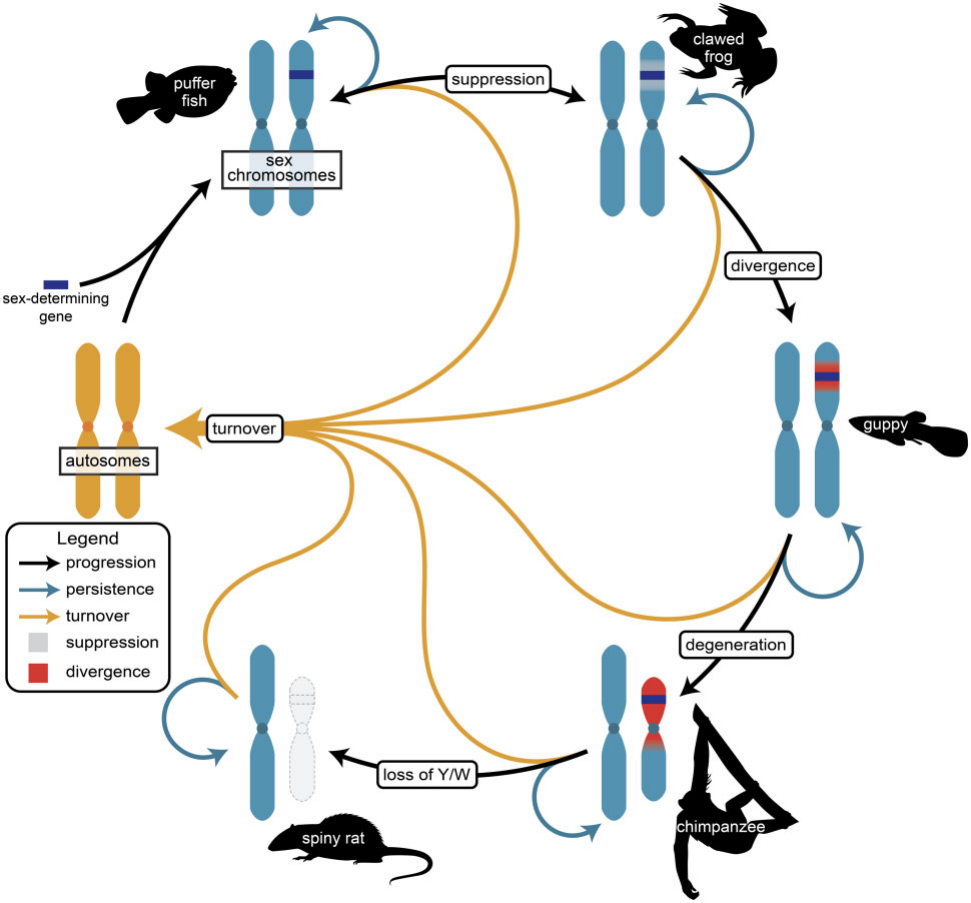
—The cycle of sex chromosome evolution. Reproduced from Furman et al. (2020). Image credit Jacelyn Shu.

Most surprisingly, studies have revealed a far greater degree of sex chromosome diversity and turnover than previously appreciated. In fact, some lineages including lizards, fish, amphibians, insects, and plants show frequent changes in the location of sex-determining genes and high rates of turnover of sex chromosomes. This led Mank and her coauthors to propose a new conceptual framework in which sex chromosome evolution is more cyclical than linear (fig. 1).

To test this cyclical theory, additional studies from a more diverse array of organisms are needed, especially from taxonomic groups that exhibit variation in sex chromosome features at the individual and population level, as well as understudied groups like fungi and protists. According to Mank, “We still don’t really understand the earliest stages of sex chromosome evolution, and I suspect this will be a major area of future focus. Additionally, our map of sex chromosomes is very sparsely populated across the tips of the tree of life, and I’m hopeful that this sampling will become much denser in the coming years, allowing for more robust comparative studies.”
